# S.W. Radiologists Association

**Published:** 1991-06

**Authors:** 


### South West Radiologists Association

### Autumn Meeting Held at Gloucester Royal Infirmary on 12th October 1990

### ADVICE TO PREMENOPAUSAL WOMEN ON BREAST SCREENING

**Dr J.B Witcombe**
Gloucester

**Trial results:** In 1986 the Forrest group recommended mammographic screening for women aged 50–64 and this recommendation was based largely on the results of the Two Counties Trial in Sweden. Since 1986 the results of the UK, Malmo and Edinburgh trials have shown less benefit than the Two Counties Trial in spite of a greater use of physical examination, more frequent screening and a greater use of two view mammography. None of these trials showed benefit for women under fifty.

**Harm-benefit balance:** There are cogent reasons why the harm-benefit (potential) balance is tilted adversely in younger women. Firstly, cancer is less common but there is a higher prevalence of fibrocystic disorders which not only cause breast lumps but interfere with the detection of neoplasia. Secondly, the sensitivity of both mammography and physical examination is lower in younger women who tend to have breasts containing more parenchyma and less fat. Thirdly the proportion of carcinoma due to *in situ* disease of uncertain clinical significance is greater in premenopausal women, and there are more borderline lesions. Finally the preclinical course of the disease is shorter. If screening should prove to be effective in young women it will need to be more frequent and sensitivity will have to be improved probably by the use of more mammographic views and greater use of physical examination. In addition sonography, needle aspiration and surgical biopsy will be needed more often. Despite thse drawbacks, screening in private centres, where screening is offered to women over 38 years of age, has expanded at the same rate as in the NHS sector.

**Family history:** Women who have a first degree relative with breast cancer have a seven-fold increased risk of developing the disease themselves at the age of 30. However this risk falls with age and at the age of forty the risk is only 30% higher than women without a family history. By the age when screening mammography may be beneficial, risk associated with a positive family history is negligible. Family history should not alter a screening strategy.

**Hormone replacement therapy:** The association of HRT and increased risk of breast cancer is still under review but it is known that HRT causes fibrocystic changes. Some radiologists recommend a "base-line" mammogram prior to HRT therapy. However the value of a "base-line" examination when non-significant change is expected seems illogical.

**Recommendations:** The DHSS advises that the screening of younger women should not be used as a source of income generation under the white paper and the NRPB and the RCR recommend that women under 50 should not be screened. Radiologists should recognise these recommendations and discourage mammographic screening in women under fifty years, the average age of the menopause.

### PERCUTANEOUS LUNG BIOPSY — A JUSTIFIABLE OUT-PATIENT PROCEDURE?

**Dr. P. Birch**
Gloucester

This paper reviewed 125 consecutive percutaneous lung biopsies performed over a 3½ year period in order to assess the efficacy and safety of the technique and its applicability to out-patients.

Numerous studies have demonstrated that most pneumothoraces are identified immediately following biopsy and most of the remainder which require treatment are detected at 1 hour. Despite this, there have been no UK reports of biopsies being performed on an out-patient basis.

After the first 20 biopsies in this series a conscious decision was taken to not admit any patient for a procedure unless there were pressing reasons for doing so.

A protocol for patient selection was presented and a standardized technique for performing the lung biopsy was described. An expiration chest X-ray is obtained on all patients 1 hour following the biopsy. Those with no evidence of pneumothorax are sent home in the company of a friend or relative with strict instructions to contact their own GP or the Radiologist concerned should they develop chest pain or shortness of breath. The remainder have a further chest X-ray at 3 hours. If the pneumothorax is small, stable and symptomless the patient is discharged with the same instructions. The remainder are admitted.

125 biopsies were carried out in 114 patients of whom 56 were out-patients. 96 biopsies were positive for malignancy giving a positive predictive value of 98.9%. The sensitivity of the test was similar for out-patient and in-patient groups. Of the 20 negative biopsies 14 were false negative results.

Pneumothorax occurred in 8 out-patients and 6 in-patients. 2 out-patients required admission for symptomatic pneumothoraces but neither required a chest drain. one in-patient required a chest drain. No out-patients were admitted after discharge. The incidence of perilesion and pleural haematoma was low and none were of any clinical significance.

The factors which place patients in a high risk category for clinically significant pneumothorax were discussed. These are patients with (i) "Severe" airways obstruction, (ii) Low arterial $pO_2$, (iii) Cavitating lesions and iv) Central lesions.

Out-patient percutaneous lung biopsy is considered a safe technique provided it is performed in a standardized manner on carefully selected patients.

### RADIOLOGIC FEATURES OF COLONIC LYMPHOMA STUDIED ON BARIUM ENEMA EXAMINATION

**S.G. Cooke, G.L. Scott, J. Virjee**
Bristol

Colonic involvement by lymphoma occurs in up to 24% of patients' studies at autopsy yet is rarely diagnosed on barium enema examination.

We have retrospectively reviewed patients with lymphoma involving the colon examined by barium enema in our department over the last 6 years. Details of patients were obtained from reviewing departmental records and by consultation with the oncology service. Five cases of lymphoma were examined with colorectal involvement. Four patients had secondary disease from non-Hodgkins lymphoma and one had primary disease. Symptoms referable to the bowel included diarrhoea, change in bowel habit, abdominal pain and weight loss.

The radiographic features on barium enema examination were analysed in detail and are discussed. The important abnormalities comprised strictures (4 cases), infiltrating plaques (2 cases), multiple nodular filling defects (2 cases), extra luminal masses (3 cases) and intraluminal mass (1 case). The most frequent site of localised involvement was the recto-sigmoid.

The distinction of strictures due to lymphoma and those due to carcinoma or other causes is important and is discussed. Using radiographic appearances alone this distinction may be difficult and can often only be made by histological analysis.

Widespread nodular lymphoma involvement may be mistaken for colonic polyposis, one of the colitides or lymphoid nodular hyperplasia. Differentiation between these entities is possible on account of the characteristic appearance of the nodules in association with other features. The relevance of lymphoid nodular hyperplasia and a possible relationship with neoplasia is discussed.

### GATED CARDIAC ISOTOPE SCANNING IN CANDIDATES FOR MAJOR VASCULAR SURGERY

**Dr S.J. Armstrong**
Bristol

The outcome of 95 patients having gated cardiac isotope scanning for estimation of left ventricular ejection fraction prior to major vascular surgery was reviewed. The aim of the study was to see whether the test altered surgical management and if it was cost-effective.

Eleven patients had low ejection fractions (taken as less than 40%). Of these 8 had aortic aneurysms, 3 of which were symptomatic. Two of these 3 patients (with EFs of 30% and 31%) had elective surgical repair and did well. One man with a very low EF of 12% did not have surgery and subsequently died of carcinoma of bronchus. Six patients with asymptomatic aortic aneurysms were treated conservatively and followed up by ultrasound. Of these one increased in size by 1.1 cm in one year and then ruptured, requiring emergency repair. Of the other 5, 3 patients have died of ischaemic heart disease and 2 are still alive.

Three patients with peripheral vascular disease had ejection fractions less than 40%. None of these were denied surgery but the surgical approach was modified, e.g. fem-fem crossover grafting instead of aorto-bifemoral grafting. Two out of these 3 patients died in the postoperative period.

The overall peri-operative mortality was 6% (5/38) in patients with ejection fractions greater than 40%, and 40% (2/5) in patients with ejection fractions less than 40%.

We conclude that a low ventricular ejection fraction is a useful predictor of increased risk of peri-operative mortality. A low ventricular ejection fraction by gated cardiac isotope scanning influences the decision to operate in asymptomatic aortic aneurysms and, when very reduced, in symptomatic aneurysms. The cost of the test is offset by potential surgical savings.

### GUEST LECTURES

### "IMAGING THE PETROUS TEMPORAL BONE"

**Peter Phelps**
Walsgrave Hospital, Coventry

The best imaging investigation of the ear is by thin section, high resolution CT in axial and coronal planes. This can show small structures in the middle ear cavity but a sound knowledge of the sectional anatomy is essential. Acquired cholesteatoma is essentially a clinical diagnosis and imaging is only necessary if there are complications. Imaging is however essential for the congenital type of cholesteatoma particularly in the petrous pyramid. Magnetic resonance has largely replaced CT for the soft tissue demonstration of masses in the petrous temporal bone and posterior cranial fossa particularly when assisted by Gadolinium enhancement. GdMRI is now the definitive investigation for acoustic neuromas although adequate preliminary screening is necessary to select the patients for GdMRI. Tumours of the middle ear cavity such as glomus tumours are best assessed by a combination of HRCT and GdMRI.

---

### NON-IMMUNE HYDROPS,

*Smoleniec & James*

West of England Medical Journal Volume 106)ii) June 1991

